# Effects of Natural and HDTMA-Br-Modified Zeolite on Cr Accumulation in *Apium graveolens* Grown in Cr(VI)-Spiked Soils

**DOI:** 10.3390/toxics14050367

**Published:** 2026-04-25

**Authors:** Evangelia Brozou, Aspasia Grammenou, Spyridon A. Petropoulos, Georgios Thalassinos, Anthoula Dimirkou, Vasileios Antoniadis

**Affiliations:** 1 Laboratory of Soil Science, Department of Agriculture Crop Production and Rural Environment, University of Thessaly, Fytokou Street, 38446 Volos, Greece; vanzelmpr@yahoo.gr (E.B.); asgrammenou@uth.gr (A.G.); gthalassinos@uth.gr (G.T.); adimirkou@uth.gr (A.D.); antoniadis@uth.gr (V.A.); 2 Laboratory of Vegetable Production, Department of Agriculture Crop Production and Rural Environment, University of Thessaly, Fytokou Street, 38446 Volos, Greece

**Keywords:** phytoremediation, soil amendments, hexavalent chromium, chromium speciation, metal uptake, soil remediation

## Abstract

Hexavalent chromium (Cr(VI)) contamination in agricultural soils poses a significant risk to environmental and food safety owing to its high mobility and acute toxicity. To investigate possible mitigation strategies, a greenhouse pot experiment was conducted using sandy loam and silty loam soils spiked with Cr(VI) at 30 mg kg^−1^ and amended with natural clinoptilolite and modified HDTMA-Br (hexadecyl-trimethyl-ammonium-bromide) zeolite, while celery (*Apium graveolens*) was cultivated to assess chromium bioavailability and plant accumulation. Hexavalent chromium concentrations declined in all treatments (up to 88.2% in sandy loam and 73.5% in silty loam), indicating progressive reduction to Cr(III), although amendment effectiveness varied by soil type. In addition, celery accumulated extremely high chromium concentrations, particularly in sandy loam soil, where root Cr(VI) reached 1776 mg kg^−1^, indicating substantial safety concerns. Translocation factor values were below 1 across treatments, indicating limited relocation of Cr from roots to shoots. In the zeolite treatments, Cr(VI) concentrations in aboveground biomass decreased; however, plant uptake was not completely inhibited. Nonetheless, the high bioaccumulation factor (Cr in plant over available Cr in soil) of as high as 34 in the Cr(VI)-amended treatment indicated an uptake potential under Cr load. We conclude that modified zeolite was successful in mitigating Cr(VI) uptake in plants. Further investigation on the effectiveness of the materials in open-field conditions is required to establish a remediation framework for Cr species.

## 1. Introduction

Soil contamination can be derived from potentially toxic elements (PTEs), such as chromium. Chromium (Cr) has received significant attention in the past decade as a major pollutant resulting from excessive anthropogenic activities [1]. The main sources of Cr contamination in the environment include mining activities, industrial applications (e.g., leather tanneries, paint production, and plating activities), industrial waste, and solid waste disposal [2]. In agricultural soils, Cr pollution is mainly attributed to the use of organic fertilizers, which have considerable Cr content [3].

Chromium (Cr) merits attention because it exists in different oxidation states that range from 0 to 6, among which trivalent (Cr(III)) and hexavalent chromium (Cr(VI)) are the most prevalent forms in the environment [4]. Cr(VI) poses the greatest environmental risk because it is acutely toxic, carcinogenic, highly mobile in the soil system, and readily bioavailable [5]. It is mainly present as anionic species, CrO_4_^−^ or Cr_2_O_7_^2−^ in the soil solution; because of its negative charge, Cr(VI) is very poorly retained by colloidal colloids [5]. Consequently, Cr(VI) is more mobile than all other cationic toxic metal(loid)s along the soil profile and readily enters plant tissues, leading to oxidative stress, destruction of cell membranes, inhibition of enzyme activity, and reduced plant growth [2]. In contrast, Cr(III) has much lower toxicity and reduced bioavailability, as it is mainly found as a cation in the form of Cr^3+^ that is strongly adsorbed onto the solid phase of soil [6]. Its strong interaction with soil colloids significantly limits its absorption by plants, although it exhibits only partial mobility under acidic soil conditions [7].

It is well known that the chemical reduction of Cr(VI) to Cr(III) is a complex process influenced by several factors, such as soil pH, redox potential, soil organic matter (SOM) content, the presence of Mn and Fe oxides, and microbial activity [8,9]. Under certain soil conditions (i.e., anoxic submerged soils or soils rich in soil organic matter (SOM) characterized by the presence of hydroxyl and phenolic groups), Cr(VI) tends to be gradually reduced to Cr(III), thereby decreasing its mobility and hazardous potential [10,11]. Conversely, in well-aerated soils or soils with low SOM levels, reduction can be more protracted; thus, Cr(VI) toxicity can be prolonged over extended periods [12].

The treatment of Cr(VI) pollution in agriculture involves various remediation strategies, among which immobilization using soil amendments has attracted particular research interest. In this context, natural zeolites have been widely used owing to their high ion exchange capacity, stable crystal structure, and ability to bind cations [13,14]. However, their effectiveness with anionic species, such as Cr(VI), is limited because the surface of the material is negatively charged [15]. For this reason, modified forms of zeolites have been developed by applying surface physical or chemical modifications, which enhance the adsorption capacity of anionic pollutants or accelerate chemical reduction processes [16,17]. These modified mineral materials have proven effective in immobilizing other hazardous elements; however, their use in growth studies is limited because of their resistance to water absorption. These materials create barriers in the soil and limit soil moisture availability, leading to water stress in plants [5].

Numerous plant species, particularly those that are tolerant to adverse environmental conditions (i.e., PTEs presence), have been studied for their phytoremediation potential [18]. The uptake of Cr by plants is influenced by various factors, such as plant species, root system physiology, interaction with ion transporters, and reduction of metal forms from Cr(VI) to Cr(III) within the roots, which mitigates its translocation to the aerial parts [19]. Among these plants, many herbaceous and edible plants have been reported to exhibit high or hyperaccumulation capability of Cr, such as *Colocasia esculenta* [20], *Vigna unguiculata* [21], *Ipomoea aquatica* [22], and *Oryza sativa* [23].

Celery (*Apium graveolens*), a vegetable with edible above-ground parts and a root system in direct contact with the soil, can accumulate PTEs in its tissues, making it a suitable candidate for studying PTE availability. In addition, it contains diverse phytochemical compounds, including alkaloids and flavonoids, which enhance its pharmaceutical and nutritional value [24,25]. The accumulation of PTEs in its edible parts highlights the need for strategies to mitigate contamination and ensure food safety. However, to the best of our knowledge, the available data in the literature on the effectiveness of zeolites and modified zeolites for the immobilization of PTEs, especially in Cr(VI), in cultivated systems and in combination with the cultivation of edible plants, remains limited despite some available studies [16,26]. In contrast to previously published work, the present study focuses on the temporal kinetics of Cr(VI) reduction, as well as on the specific bioaccumulation (BAF) and translocation factors (TFs) of Cr species in celery tissues, which have not been previously investigated.

Therefore, this study aimed to investigate:

(i) The temporal evolution of hexavalent chromium (Cr(VI)) concentration in two different soil types (sandy loam and silty loam);
(ii) The effect of natural and modified zeolites on bioavailability of Cr(VI) and its reduction to Cr(III);
(iii) The accumulation of two species of Cr in the tissues and roots of celery.

This work contributes to the understanding of the mechanisms of Cr binding and transformation in soil and the practical application of low-cost materials for reducing soil contamination and increasing the safety of agricultural products.

## 2. Materials and Methods

### 2.1. Experimental Design

A pot experiment was conducted under greenhouse conditions from October to February using two soil types: sandy loam and silty loam, with the initial physicochemical properties summarized in Table 1. Greenhouse conditions were unheated and relied on natural sunlight. Throughout the experimental period, soil moisture was kept constant at of 65% water-holding capacity and greenhouse temperature was maintained within a range of 25–35 °C.

Four treatments were applied for each soil type:

- Control (C): soil with no further amendments;
- Cr(VI): soil + 30 mg kg^−1^ Cr(VI) (as CrO_3_);
- Cr(VI) + natural clinoptilolite zeolite (Cr(VI) + Z);
- Cr(VI) + HDTMA-Br-modified clinoptilolite zeolite (Cr(VI) + MZ)

The experiment was based on a completely randomized design with three replicates per treatment, resulting in 24 pots. Each pot contained 2 kg of soil. Natural clinoptilolite zeolite was supplied by S&B Industrial and applied at a soil-to-adsorbent ratio of 200:1 (*w*/*w*). Its key physicochemical properties were as follows: specific area 30.7 m^2^ g^−1^; cation exchange capacity (CEC) 235 cmol_c_ kg^−1^; and zero point of charge (ZPC) of 6.8. Modified zeolite (MZ) was prepared by treating natural zeolite with 0.05 N HDTMA-Br (hexadecyl-trimethyl-ammonium-bromide; 3 g zeolite:25 mL solution) for 24 h under agitation, followed by rinsing to stable conductivity and oven-drying. This modification is expected to alter the surface properties of zeolite, due to the formation of an organic cation layer to the external surface [27]. The detailed composition and additional physicochemical characteristics of natural zeolite are provided in Table S1 (Supplementary Material).

Celery seeds were directly sown into 2 L pots to ensure uniform germination across all plots, allowing precise timing of Cr(VI) application and better control of the experimental conditions. The soil was spiked with a total of 30 mg kg^−1^ Cr(VI) (as CrO_3_) in three equal doses at 15, 20, and 22 days after sowing. A 10,000 ppm Cr(VI) stock solution was prepared by dissolving CrO_3_ in deionized water, and 2 mL of this solution was applied per pot for each dose. This application was used to allow gradual accumulation of Cr in the soil and to avoid potential toxicity from a single large dose. Prior to Cr(VI) application, soils were thoroughly mixed with the respective zeolite amendments (when applicable) to ensure uniform distribution of the adsorbent. After each Cr(VI) application, soils were manually homogenized within the pot. This concentration of Cr(VI) was chosen based on literature to ensure measurable effects in the experiment. There are not set regulatory limits for Cr(VI) in soil in the European Directive; regulations typically refer to total Cr. Fertilization was applied once, 10 days after sowing, using a slow-release fertilizer (Entec 26-0-0 + 13S) at 0.5 g per pot (0.25 g kg^−1^ soil). This rate was based on typical recommendations for pot experiments to ensure adequate and consistent nitrogen availability during plant growth. The pots were regularly irrigated to maintain soil moisture near field capacity (i.e., the soil water content after excess water had drained and drainage had ceased), and were repositioned weekly to minimize positional negative effects due to any differences in light, shade, and temperature in the greenhouse.

### 2.2. Soil and Plant Analyses

During the experiment, soil samples were collected on days 46, 56, and 63 after sowing to measure exchangeable Cr(VI). At each sampling time, composite soil samples, ca. 50 g per pot, were taken from three random points within each pot using a stainless cylinder inserted to the full pot depth. Only a small fraction of soil was removed each sampling time from the same pots throughout the experiment, ensuring minimal disturbance of the system. At the end of the experiment, soil samples were collected for the determination of pseudo-total Cr, extractable Cr(III), and exchangeable Cr(VI), while the plant samples were harvested and separated into roots and aboveground parts, for the measurements of Cr(III) and Cr(VI). The plant samples were washed to remove foreign particles, dried in an oven at 70 °C for 48 h, and ground into a fine powder. The soil samples were air-dried, sieved through a 2 mm sieve, and homogenized before analysis. The physicochemical characteristics of the soil samples were determined using established methods. The pH and electrical conductivity (EC) were measured in a soil–water suspension (1:5; *w*/*v*). The CaCO_3_ content was determined using a Bernard calcimeter (Pobel, Madrid, Spain), soil texture was determined using a Bouyoucos hydrometer (Pobel, Madrid, Spain), and SOM content was evaluated using the modified Walkley–Black method. Pseudo-total chromium in the soil was determined after aqua regia digestion (HCl:HNO_3_, 75:25, *w*/*w*) at 180 °C for 2 h and measured by atomic absorption spectrophotometry (Perkin Elmer 3300; Perkin Elmer, Shelton, CT, USA). Measurements were performed at wavelength 357.9 nm, using an air-acetylene flame under standard operating conditions. Calibration curves were constructed using Cr standard solutions in the range of 0.16–10 mg L^−1^, with a coefficient of determination R^2^ ≥ 0.995. Analytical quality was ensured using sample blanks and quality control samples (QCS) in each analytical batch. The limit of detection (LOD) for Cr was 0.36 mg L^−1^. Available Cr(III) was extracted using DTPA (diethylenetriaminepentaacetic acid) at pH 8.3, according to Lindsay and Norvell [28], while exchangeable Cr(VI) was extracted with 0.01 M KH_2_PO_4_ and determined colorimetrically using the diphenylcarbazide method, with absorbance measured at 540 nm (Shimadzu UV-Vis 120-01; Shimadzu Corporation, Kyoto, Japan). Calibration was performed using Cr(VI) standard solutions over an appropriate concentration range, and reagent blanks were used for baseline correction.

For plant samples, total Cr was determined by dry ashing the sample (1 g) at 520 °C for 24 h, followed by dissolving the residue with 20 mL of 20% HCl solution, and measuring the Cr content using a Perkin Elmer 3300 atomic absorption analyser (Perkin-Elmer 3300 Spectrophotometer, Perkin Elmer, Nor-walk, CT, USA). Cr(VI) was quantified using the diphenylcarbazide method, with absorbance measured at 540 nm.

The concentration of Cr(III) in plant tissues was calculated indirectly by subtracting the Cr(VI) from the total Cr. All reagents used in this study were of analytical grade (Merck, Germany), all solutions were prepared using deionized water.

### 2.3. Indices

To assess the efficiency of Cr uptake from soil and its translocation within plant tissues, bioaccumulation factor (BAF) and translocation factor (TF) were determined. As proposed by Kikis et al. [29], these factors are defined as follows, based on the corresponding element-specific soil extractable fraction:

Bioaccumulation factor (BAF):

(1) $$\mathrm{BAF}\;=\;\left(\frac{\mathrm{Concentration}\;\mathrm{of}\;\mathrm{PTEs}\;\mathrm{in}\;\mathrm{aerial}\;\mathrm{biomass}\;(\mathrm{mg}\;{\mathrm{kg}}^{-1}\;\mathrm{dry}\;\mathrm{weight})}{\mathrm{Extractable}\;\mathrm{concentration}\;\mathrm{of}\;\mathrm{PTEs}\;\mathrm{in}\;\mathrm{soil}\;(\mathrm{mg}\;{\mathrm{kg}}^{-1}\;\mathrm{dry}\;\mathrm{soil})}\right),$$

Translocation factor (TF):

(2) $$\mathrm{TF}\;=\;\left(\frac{\mathrm{Concentration}\;\mathrm{of}\;\mathrm{PTEs}\;\mathrm{in}\;\mathrm{aerial}\;\mathrm{biomass}\;(\mathrm{mg}\;{\mathrm{kg}}^{-1}\;\mathrm{dry}\;\mathrm{weight})}{\mathrm{Concentration}\;\mathrm{of}\;\mathrm{PTEs}\;\mathrm{in}\;\mathrm{roots}\;(\mathrm{mg}\;{\mathrm{kg}}^{-1}\;\mathrm{dry}\;\mathrm{weight})}\right),$$

### 2.4. Statistical Analysis

All measurements were performed in triplicate. The data were analyzed using one-way analysis of variance (ANOVA) for each soil type at a significance level of *p* < 0.05. Duncan’s multiple range test was applied for the comparison of means. Statistical analyses were performed using the IBM SPSS version 30 (IBM Corp., Armonk, NY, USA) statistics software.

## 3. Results

### 3.1. Pseudo-Total and Available Chromium in Soil

**Figure 1:**
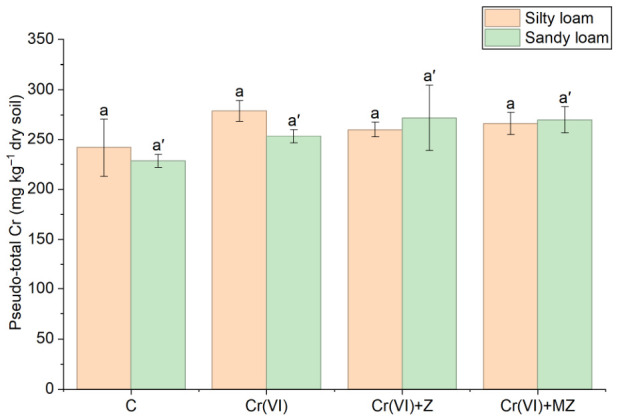
Pseudo-total Cr concentrations (mg kg^−1^ dry soil) in soils cultivated with *Apium graveolens* under different treatments C (control), Cr(VI) (30 mg kg^−1^ as CrO_3_), Cr(VI) + Z (natural zeolite), and Cr(VI) + MZ (modified zeolite). Bars represent mean values ± SE. Different lowercase letters indicate statistically significant differences among treatments within the silty loam soil, whereas different lowercase letters with primes indicate statistically significant differences among treatments within the sandy loam soil (*p* < 0.05, Duncan’s test).

**Figure 2:**
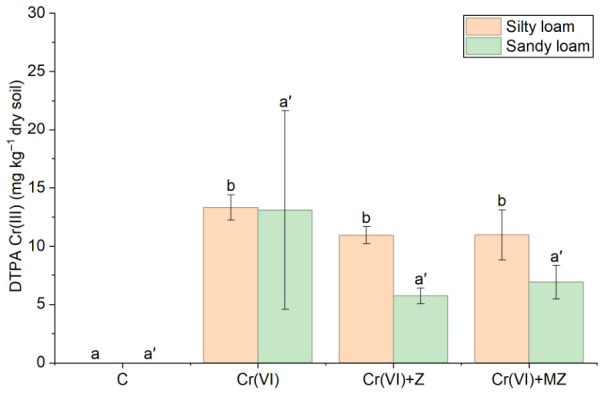
DTPA-extractable Cr(III) concentration (mg kg^−1^ dry soil) at the final sampling day in soils under the different treatments C (control), Cr(VI) (30 mg kg^−1^ as CrO_3_), Cr(VI) + Z (natural zeolite), and Cr(VI) + MZ (modified zeolite). Bars represent mean values ± SE. Different lowercase letters indicate statistically significant differences among treatments within silty loam soil, whereas different lowercase letters with primes indicate statistically significant differences among treatments within sandy loam soil (*p* < 0.05, Duncan’s test).

The pseudo-total concentration of Cr at all treatments did not differ significantly, regardless of the soil type (Figure 1). Likewise, DTPA-extractable Cr(III) did not show any notable differences between the treatments applied, i.e., Cr(VI), Cr(VI) + Z, and Cr(VI) + MZ, in both the sandy loam and silty loam soils (Figure 2).

### 3.2. Temporal Dynamics of Cr(VI) Concentration in Soils Cultivated with Celery Under Different Soil Amendments

**Figure 3:**
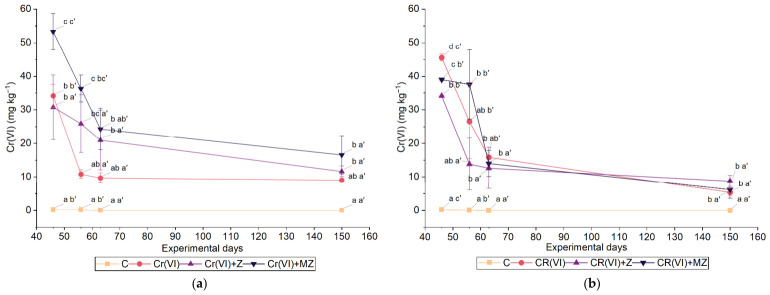
Temporal variation in Cr(VI) concentration (mg kg^−1^ dry soil) in (**a**) silty loam and (**b**) sandy loam soils cultivated with *Apium graveolens* under control (C), Cr(VI) (30 mg kg^−1^ as CrO_3_), Cr(VI) + Z (natural zeolite), and Cr(VI) + MZ (modified zeolite) treatments. Values are presented as mean ± SΕ. Different lowercase letters indicate significant differences among treatments within the same sampling time, whereas different lowercase letters with primes indicate significant differences among sampling dates within the same treatment (at *p* < 0.05 according to one-way ANOVA).

As shown in Figure 3, Cr(VI) concentrations decreased over time in both soil types. During the initial sampling of the sandy loam soil, the lowest Cr(VI) content was measured with zeolite treatment, followed by the application of the modified zeolite. In the silty loam, significant differences among amendments were observed during the first sampling, with the Cr(VI) + MZ treatment exhibiting significantly higher Cr(VI) concentrations than all other Cr-amended treatments. In the sandy loam soil, in the first sampling, the Cr(VI) treatment had the highest concentration (45.6 mg kg^−1^). When zeolite was applied, Cr levels decreased significantly by 15% with Cr(VI) + MZ and by 26% with Cr(VI) + Z compared to the Cr(VI) treatment (Figure 3). Notably, in the silty loam, at the first sampling, the Cr(VI) + MZ treatment exhibited the highest concentration (53.3 mg Cr(VI) kg^−1^), considerably higher by 36% and 44% than Cr(VI) and Cr(VI) + Z, respectively. This trend continued in the second sampling, with the Cr(VI) + MZ treatment being significantly higher by 29% and 63% than Cr(VI) + Z and C(VI), respectively.

During the last sampling, in both soils, no statistically significant differences were recorded among the Cr(VI), Cr(VI) + Z, and Cr(VI) + MZ treatments. In the sandy loam soil, the percentage of decrease in the Cr(VI) concentration from the first to the last sampling was 88.2% in the Cr(VI) treatment, 74.4% at Cr(VI) + Z, and 84.1% at Cr(VI) + MZ. Correspondingly, in the silty loam soil, the percentage of decrease reached 73.5%, 62.1%, and 69.0% at Cr(VI), Cr(VI) + Z, and Cr(VI) + MZ, respectively (Figure 3).

### 3.3. Chromium Accumulation in Celery

**Figure 4:**
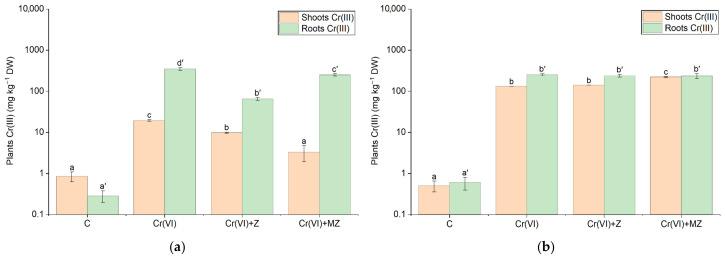
Cr(III) concentration (mg kg^−1^ dry weight) in the shoots and roots of *Apium graveolens* cultivated in (**a**) silty loam and (**b**) sandy loam soils under control (C), Cr(VI) (30 mg kg^−1^ as CrO_3_), Cr(VI) + Z (natural zeolite), and Cr(VI) + MZ (modified zeolite) treatments. Values represent mean ± SE. Different lowercase letters indicate significant differences among treatments in shoots, whereas different lowercase letters with primes indicate differences among treatments in roots (*p* < 0.05, Duncan’s test). Y-axis is presented on logarithmic (log10) scale.

Our data demonstrate different behaviors based on Cr oxidation levels and soil types. On sandy loam, in the aboveground biomass, Cr(III) accumulated at its highest rate at the Cr(VI) + MZ level (i.e., 221 mg kg^−1^), approximately a 2-fold increase compared to the other treatments (Figure 4b). In contrast, on silty loam, Cr(III) in the Cr(VI) treatment exhibited the highest accumulation (19.5 mg kg^−1^), significantly higher than in all other treatments (Figure 4a). In both soils, Cr(III) accumulation at the control treatment was kept at around 0.1 mg kg^−1^, which was the lowest among treatments. In contrast to the aboveground biomass, Cr(III) root concentration did not differ significantly across treatments in the sandy loam soil, whereas in the silty loam, the Cr(VI) treatment exhibited the highest accumulation, i.e., 350 mg kg^−1^, 40% significantly higher than that at Cr(VI) + MZ, and 5.5-fold higher than Cr(VI) + Z (Figure 4a).

**Figure 5:**
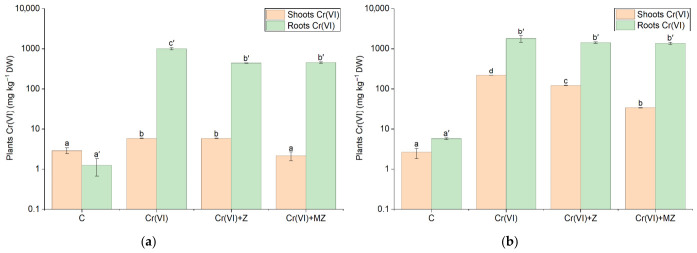
Cr(VI) concentration (mg kg^−1^ dry weight) in the shoots and roots of *Apium graveolens* cultivated in (**a**) loam and (**b**) sandy loam soils under control (C), Cr(VI) (30 mg kg^−1^ as CrO_3_), Cr(VI) + Z (natural zeolite), and Cr(VI) + MZ (modified zeolite) treatments. Values are presented as mean ± SE. Different lowercase letters indicate significant differences among treatments in shoots, whereas different lowercase letters with primes indicate differences among treatments in roots (*p* < 0.05, Duncan’s test). Y-axis is presented on logarithmic (log10) scale.

Contrary to Cr(III), Cr(VI) accumulation in sandy loam soil was mostly observed in the Cr(VI) treatment. Specifically, in the aboveground biomass, hexavalent Cr levels were 216 mg kg^−1^ in the Cr(VI) treatment, 44% higher than those in the Cr(VI) + Z and 84% higher than those in the Cr(VI) + MZ treatments (Figure 5b). In roots, hexavalent Cr was accumulated at levels as high as 1776 mg kg^−1^ in the Cr(VI) treatment; however, these concentrations did not differ statistically from those in all other treatments (Figure 5b). In the silty loam, Cr(VI) accumulation in the aerial parts of celery was highest in the Cr(VI) and Cr(VI) + Z treatments, with an accumulation of 5.83 mg kg^−1^, which was twice the level of hexavalent Cr in the Cr(VI) + MZ and control treatments (Figure 5a). In roots, Cr(VI) was accumulated at a level as high as 1005 mg kg^−1^ in the Cr(VI) treatment and was notably higher by 55% than in the Cr(VI) + Z and Cr(VI) + MZ treatments (Figure 5a).

### 3.4. Bioaccumulation (BAF) and Translocation (TF) Indices

**Figure 6:**
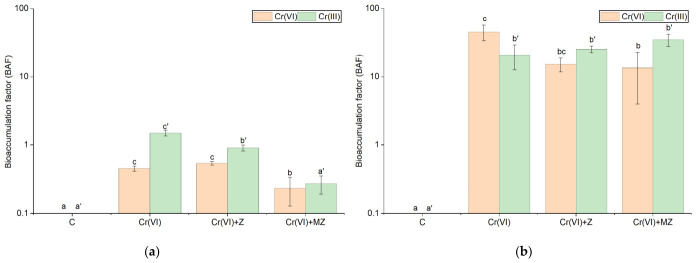
Bioaccumulation factor (BAF) of Cr(III) and Cr(VI) in *Apium graveolens* cultivated in (**a**) silty loam and (**b**) sandy loam soils under control (C), Cr(VI) (30 mg kg^−1^ as CrO_3_), Cr(VI) + Z (natural zeolite), and Cr(VI) + MZ (modified zeolite) treatments. Values are presented as mean ± SE. Different lowercase letters with primes indicate significant differences among treatments for the BAF of Cr(III), whereas different lowercase letters indicate significant differences among treatments for the BAF of Cr(VI) (*p* < 0.05, Duncan’s test). Y-axis is presented on logarithmic (log10) scale.

When the BAF and TF values were calculated for Cr(III), no statistically significant differences were observed across treatments in sandy loam soil; however, the BAF values recorded ranged from 21 at Cr(VI) to 35 at Cr(VI) + MZ. In the silty loam, BAF values were the highest in the Cr(VI) treatment (BAF = 1.5), whereas the other treatments were significantly lower, i.e., 0.91 at Cr(VI) + Z and 0.27 at Cr(VI) + MZ (Figure 6a).

**Figure 7:**
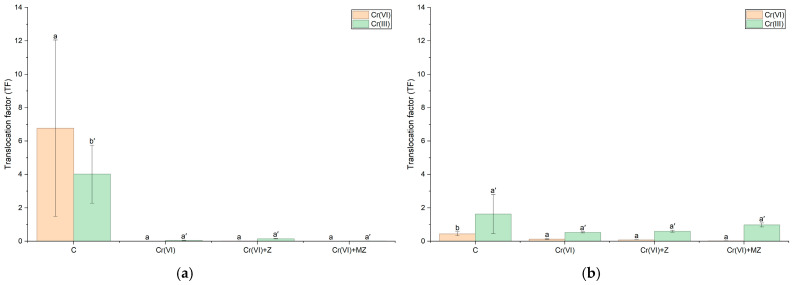
Translocation factor (TF) of Cr(III) and Cr(VI) in *Apium graveolens* cultivated in (**a**) silty loam and (**b**) sandy loam soils under control (C), Cr(VI) (30 mg kg^−1^ as CrO_3_), Cr(VI) + Z (natural zeolite), and Cr(VI) + MZ (modified zeolite) treatments. Values are presented as mean ± SE. Different lowercase letters with primes indicate significant differences among treatments for the TF of Cr(III), whereas different lowercase letters indicate significant differences among treatments for the TF of Cr(VI) within each soil type (*p* < 0.05, Duncan’s test).

Regarding Cr(VI), the TF values did not differ significantly from each other in both soil types and remained consistently below unity (Figure 7). In sand, TF values ranged from 0.13 at Cr(VI) to as low as 0.03 at Cr(VI) + MZ, while in the silty loam, they were even lower, not exceeding 1.3 × 10^−2^ (Cr(VI) + Z) (Figure 7a). With respect to the BAF values, the Cr(VI) treatment showed a significantly higher BAF value than the other treatments, reaching 35 in sandy loam (Figure 6b). As in the silty loam, in the Cr(VI) and Cr(VI) + Z treatments, BAF values (0.45 and 0.54, respectively) were significantly higher than those of the other treatments, being twice as high as that at Cr(VI) + MZ (BAF = 0.23) (Figure 6a).

## 4. Discussion

The results of the present study show clear differentiation in the dynamics and impact of the two studied chromium species (Cr(VI) and Cr(III)) in soil and plants following soil amendments with natural clinoptilolite zeolite (Z) and HDTMA-Br-modified clinoptilolite zeolite (MZ). The absence of significant differences in pseudo-total Cr in the soils amended with the two types of zeolites indicates that the application of these amendments did not affect the overall Cr load in the soil. This may also be attributed to the contribution of the natural background levels of the soil, which may mask differences among treatments. Similar findings have been reported by Cadar et al. [30], who reported no changes in pseudo-total Cr concentration after the addition of zeolite. In contrast, Cr speciation and its interactions with soil after amendment appear to play a significant role regarding its available fraction. Several studies have highlighted the high adsorption capacity of Cr cations by materials with elevated cation exchange capacity (CEC), such as zeolites [31].

The observed decline in Cr(VI) concentrations over time across all treatments is consistent with a progressive reduction of Cr(VI) to the less toxic Cr(III), a process likely driven by the soil’s inherent reductive capacity [32]. The initial effectiveness of zeolite in sand, with a significant reduction in Cr(VI) from the first sampling, could be associated with the high specific surface area and ion exchange capacity of this material, as well as possible electrostatic interactions between the anionic species of Cr(VI) and positively charged surface sites, especially under suitable pH conditions [33]. In addition, zeolite application has been shown to improve soil structure [34] and enhance water capacity, reducing the risk of excessive saturation in the root zone [35]. These changes may create favorable conditions for increased microbial biomass and activity, and consequently could enhance the biotic reduction of Cr(VI) to Cr(III), a mechanism that has been highlighted in corresponding studies [36,37]. In contrast to the results observed in sandy loam soil, the MZ treatment in silty loam showed higher Cr(VI) concentrations than the Cr(VI) + Z, likely due to the chemical nature of the modifier used. HDTMA, as a cationic surfactant, exchanges with the inorganic cations of clinoptilolite, forming a double layer that gives the modified zeolite the ability to adsorb cations, non-polar organic compounds, and anions (i.e., CrO_4_^−^) [27]. Nevertheless, as adsorption model parameters are sensitive to regression choices [38], the precise interaction between MZ and soil anions remains a complex process. However, in clay-rich soils, coexisting anions such as sulfate, bicarbonate, and phosphate can compete for anion exchange sites, potentially displacing Cr(VI) into the soil solution and leading to higher levels of soluble Cr(VI) [39,40]. Furthermore, although soil pH was not directly measured, the literature supports that at higher pH, the capacity of HDTMA-modified zeolite may decrease. Under alkaline conditions, the enhanced electronegativity of clay and oxide surfaces could increase the electrostatic repulsion of CrO_4_^−^, thereby keeping the anion in the soil solution [41]. The Cr(VI) concentrations in the final sampling indicated no significant differences between treatments, suggesting that over time, stabilization mechanisms likely prevailed regardless of modification, with the higher decreased Cr(VI) rates being recorded in the sandy loam soil (up to a 88% decrease in the last sampling compared to the initial). The variation in reduction rates between the two soil types highlights the key role of important soil properties, such as texture and clay content.

Regarding the distribution of Cr species in plants, the high accumulation of Cr(VI) in roots, especially in sandy loam soil (1776 mg kg^−1^), indicates intense initial uptake but with limited subsequent movement in the aboveground biomass, as confirmed by TF < 1. TF values less than unit indicate the capability of plants to confine PTEs in underground biomass [28]. This is the case when, in excluder plants, roots act as a primary barrier preventing the accumulation of toxic Cr(VI) in leaves, thus excluding the toxic metal from adversely affecting vital plant physiological functions. Similar root accumulation has been observed in experiments with *Pteris vittata*, where it was reported that over 96–99% of Cr(VI) was concentrated in the roots under an exposure of as high as 100 μM Cr(VI) (equivalent to 5.20 mg L^−1^), with very little transfer to the aboveground parts, and with almost all of the transferred Cr upwards towards the leaves being reduced to Cr(III) in the roots [42]. Although only Cr(VI) was applied to the soil, a notable presence of Cr(III) above the control levels was detected in plant tissues. These findings suggest that reduction of Cr(VI) to Cr(III) may occur not only in the soil but can also continue in the rhizosphere and/or plant tissues. Cr(VI) is absorbed by plants mainly through phosphate and sulfate transporters due to its structural similarities with P and S [43]. Thereafter, ferrous reductase enzyme activity in the roots is likely involved in its conversion to Cr(III), which accumulates and is immobilized mainly in the vacuoles of root cells [44].

The impact of the additives is clearly reflected in the Cr concentrations in plant tissues and is inextricably linked to the bioavailability of the different forms of Cr. In the sand, despite the reduction in Cr(VI) in the soil solution, root concentrations reached up to 1776 mg kg^−1^. There was no significant decrease in the treatments under Z and MZ, suggesting that the addition of materials was not sufficient to substantially limit root uptake under high Cr(VI) loads. At the same time, the above-ground parts showed Cr(VI) concentrations of up to 216.3 mg kg^−1^, indicating that in soils with low buffering capacity, plant uptake remains high. However, both materials significantly reduced Cr(VI) transport to above-ground parts, with MZ being more effective, possibly due to its enhanced anion exchange capacity and better a higher potential for binding of CrO_4_^2−^ ions in the soil solution [45]. It is acknowledged, however, that without equilibrium adsorption testing and defensible regression analysis [38], these binding mechanisms remain an interpretation based on observed bioavailability trends. In the silty loam, the presence of modified zeolite (MZ) led to decreased Cr species concentrations in the above-ground parts (~2.9 mg kg^−1^ at Cr(VI) + MZ, compared to 5.9 mg kg^−1^ in the treatments of Cr(VI) and Cr(VI) + Z), indicating the higher effectiveness of MZ in immobilizing CrO_4_^2−^ anions in the clay environment. For Cr(III), both materials significantly decreased absorption in both shoots and roots, indicating effective immobilization of this Cr species. These findings are consistent with those of previous studies. Radziemska et al. [13] reported that soil amendments, including zeolite, in Cr(III)-contaminated soil decreased plant Cr uptake by spring barley and maize. From a food safety perspective, it is worth noting that the literature reports that physiological Cr concentrations in edible vegetables are generally very low (0.02–0.2 mg per kg DW), and exceeding this range is considered a potential food risk [46]. Our findings indicate that the Cr levels in the above-ground parts of celery, especially in the sand, far exceeded the expected physiological concentrations. This highlights the significance of selecting suitable plant species and soil amendment materials while considering the physicochemical characteristics of the soil to minimize the transfer of PTEs into the food chain.

High Cr(VI) BAF values in the treatments without zeolite amendment in the sand indicate increased net uptake in a low-buffering capacity environment, which implies increased environmental and dietary risk. Most available literature data mainly concern related species of the *Apiaceae* family (e.g., *Daucus carota*), reporting a median BAF value of 2.51 [47]. Soil properties, such as texture and organic carbon content, have been consistently recognized as important determinants of Cr availability; coarse-textured soils with low SOM exhibit higher bioavailability and plant uptake than fine-textured soils with elevated SOM content [48]. In our silty loam soil, BAF values were significantly lower than those in the sand, confirming stronger adsorption and reduction processes limiting plant availability. Accordingly, BAF values for Cr(III) support this interpretation. In the sand, high BAF values (20.8–34.8), despite a stable soil DTPA form, indicate that plant Cr(III) concentration does not simply reflect available Cr(III) in the soil but is a product of Cr(VI) transformation to the reduced Cr(III) species after uptake. In contrast, in the silty loam soil, the lower BAF values (<1.5) suggest limited net accumulation relative to soil concentration, which is consistent with stronger geochemical control.

## 5. Conclusions

This study demonstrated that soil texture is a key factor controlling the dynamics, speciation, and plant uptake of Cr. A progressive decrease in soil available Cr(VI) concentrations over time, resulting from its gradual reduction to Cr(III), was observed in both soils, thereby affecting the bioavailability of the element. The application of natural and modified zeolite amendments did not significantly affect the pseudo-total Cr concentration; however, notable changes in available soil Cr(VI) concentration and behavior were observed within each soil type, indicating the effectiveness of these materials in stabilizing and transforming Cr(VI). Despite these beneficial effects, the materials did not completely inhibit plant uptake under stress conditions. *Apium graveolens* exhibited significant Cr accumulation, mainly in the root system (TF < 1), confirming limited translocation to aboveground parts. However, specific treatments showed elevated Cr concentrations in edible plant parts, highlighting a potential food risk in contaminated agricultural soils. The findings of this study contribute to the elucidation of Cr binding and transformation mechanisms at the soil–plant interface; however, because the experiment was conducted under controlled conditions, we consider it an initial step towards addressing the issue of Cr mobility from soil to plants. Hence, further investigations under field conditions in real Cr-contaminated agricultural areas are needed to confirm the effectiveness of the tested materials.

## Figures and Tables

**Table 1:** Physicochemical properties of soils.

E.C	pH	CaCO_3_	SOΜ	OC	Soil Texture			Soil
μS cm^−1^		%	%	%	% Sand	% Clay	% Silt	Classification
178.3	8.35	12.0	1.3	0.7	78.4	6.4	15.2	Sandy loam
240.8	8.03	11.7	0.9	0.6	34.8	14.4	50.8	Silty loam

## Data Availability

The original contributions presented in this study are included in the article. Further inquiries can be directed to the corresponding author.

## Supplementary Materials

The following supporting information can be downloaded at https://www.mdpi.com/article/10.3390/toxics14050367/s1, Table S1: Physicochemical properties of zeolite [49,50,51].

## Abbreviations

The following abbreviations are used in this manuscript:

BAF	Bioaccumulation Factor
Cr(VI)	Hexavalent Chromium
Cr(III)	Trivalent Chromium
DTPA	Diethylenetriaminepentaacetic acid
HDTMA-Br	Hexadecyl Trimethyl Ammonium Bromide
MZ	Modified Zeolite
PTEs	Potentially Toxic Elements
SOM	Soil Organic Matter
TF	Translocation Factor
