# Supplementary material for: Effects of Natural and HDTMA-Br-Modified Zeolite on Cr Accumulation in Apium graveolens Grown in Cr(VI)-Spiked Soils
Source: Toxics. 2026 Apr 25;14(5):367. doi: 10.3390/toxics14050367 (PMC13211114; doi:10.3390/toxics14050367)
Supplement: Supplementary file 1 [file toxics-14-00367-s001.zip › Table S1.pdf]

Table S1. Physicochemical properties of Zeolite

Property	Value	Method
Specific area (m^2g^{-1})	30.7	[49]
Cation exchange capacity (CEC) ($cmol_c Kg^{-1}$)	235	[50]
Zero point of charge (ZPC)	6.8	[51]
Chemical composition		
SiO_2 (%)	66.98	Manufacturer
Al_2O_3 (%)	13.31	Manufacturer
FeO (%)	0.98	Manufacturer
MgO (%)	0.87	Manufacturer
CaO (%)	3.43	Manufacturer
Na_2O (%)	0.53	Manufacturer
K_2O (%)	0.78	Manufacturer
